# Dental Trauma in Martial Arts Practitioners: A Cross-Sectional Study in a Capital City of Southeastern Brazil

**DOI:** 10.3390/dj14080510

**Published:** 2026-08-11

**Authors:** Gabriela Almeida Souza Leão Simonton, Pamela Barbosa dos Santos, Lorrayne Cesario Maria, Gabriel Pereira Nunes, Maria Helena Monteiro de Barros Miotto

**Affiliations:** 1 Department of Postgraduate Studies in Dental Sciences, Federal University of Espírito Santo, Av. Marechal Campos, 1468, Maruípe, Vitória 29043-900, ES, Brazil; 2 Department of Prosthodontics and Periodontology, Piracicaba Dental School, University of Campinas, Avenida Limeira, 901, Piracicaba 13414-903, SP, Brazil; 3 Department of Postgraduate Studies in Public Health, Federal University of Espírito Santo, Av. Marechal Campos, 1468, Maruípe, Vitória 29043-900, ES, Brazil

**Keywords:** dental trauma, prevalence, sports dentistry, martial arts, public health

## Abstract

**Background/Objectives:** Dental trauma is recognized as a global public health problem due to its prevalence and psychosocial consequences. This study aimed to verify the prevalence of traumatic dental injuries in practitioners of combat sports and whether they were associated with sociodemographic and sports variables in a capital city in southeastern Brazil. **Methods:** This cross-sectional observational study included 284 athletes over 18 years of age from 17 academies that offered combat sports. Data collection was performed by a single examiner through individual interviews and a questionnaire addressing sociodemographic profile, access to oral health services, sport modality, experience with dental trauma during combat sports, mouthguard use, and guidance on dental injuries. Descriptive analyses, frequency tables, the Chi-square test (*p* < 0.05), and Odds Ratio were used. **Results:** Most participants were male (*n* = 203; 71.48%), aged up to 25 years (*n* = 75; 26.41%). The most practiced sports were Jiu-Jitsu (*n* = 79; 27.82%) and Kickboxing (*n* = 77; 27.11%). Among participants who suffered dental trauma (*n* = 19; 6.69%), single episodes predominated (*n* = 9; 47.37%), enamel fractures were most frequent (*n* = 7; 36.84%), and anterior teeth were the most affected (*n* = 9; 47.36%). Most were not using a mouthguard at the time of trauma (*n* = 15; 78.95%), although they considered mouthguard use important (*n* = 229; 80.63%), and most had not received guidance on dental trauma (*n* = 237; 83.45%). Age group was significantly associated with dental trauma (*p* = 0.016; OR = 3.33; 95% CI: 1.167–9.524). **Conclusions:** The prevalence of dental trauma among fighters was low. Mouthguard adherence was inconsistent, post-trauma management was inadequate, and most respondents had never received guidance on dental trauma. Public health policies should strengthen awareness and prevention of dental injuries in sports.

## 1. Introduction

Among facial injuries, traumatic dental injuries (TDIs) and dentoalveolar trauma stand out, classified as the second most prevalent oral condition and the fifth most common injury in the world, characterizing a serious public health problem [1]. Traffic accidents, falls, situations of violence, and sports activities are examples of etiological factors listed for trauma [2,3]. These injuries affect the quality of life of individuals in a multifactorial way because they interfere with oral function and aesthetics, in addition to generating emotional discomfort [4,5]. Normally, the upper anterior teeth are the most involved, which leads to consequences [6,7].

Currently, there is a continuous increase in the adoption of physical activity practices in gyms, clubs, courts, and outdoor spaces, reflecting a lifestyle focused on health promotion. Furthermore, sports practice is associated with psychological well-being, expressed by higher levels of self-esteem and life satisfaction, and lower rates of depression, anxiety, and stress, as well as better social outcomes, including self-control, pro-social behavior, and interpersonal communication [8]. However, despite the benefits, the occurrence of adverse factors, such as sports injuries, is inevitable [9]. Therefore, dental trauma is considered the intersection point between dentistry and sports and can affect athletes or practitioners of all ages and skill levels during training and competitions, impacting their performance [10]. Previous studies demonstrate differences in injury distribution according to age, suggesting that injury risk depends on various factors such as the sport practiced, athlete experience, and training duration [3,10,11,12,13,14,15,16,17,18].

In contact sports, the incidence of dental trauma is significantly higher than in non-contact sports (various forms of martial arts fall into this category, such as boxing, jiu-jitsu and taekwondo) [19]. Notably, there are more injuries in combat sports [20]. The International Dental Federation (FDI) classifies them as high-risk sports due to the frequency of impacts to the facial region [21]. It is estimated that between 18% and 30% of traumas suffered by athletes involve the oral cavity [22]. Given this scenario, on a global scale, there is a growing development of protocols aimed at standardizing the notification, counting, and treatment of dental trauma. If all of them were recorded, the number reported would be more significant and would thus indicate a greater need to implement public policies involving prevention programs and awareness campaigns [23,24].

Therefore, dental traumas (DTs) constitute a recurring complication among sports practitioners, with a prevalence ranging from 0.71% to 60%, depending on the characteristics of each sport and local cultural factors [1]. The epidemiological understanding of these injuries is still limited, but fundamental to supporting strategies, guiding public policies, and improving dental care protocols in sports environments. In this sense, identifying the characteristics and frequency of dental traumas contributes to the formulation of measures mainly related to promoting the oral health of athletes. Thus, the present study aimed to verify the prevalence of DTs in wrestlers from the city of Vitória, Espírito Santo, where there are still no scientific records on the subject, thus justifying the relevance and originality of this research.

## 2. Materials and Methods

This research was approved by the Research Ethics Committee of the Federal University of Espírito Santo (CAAE: 81697724.0.0000.5060) and prepared in accordance with the guidelines of Strengthening the Reporting of Observational Studies in Epidemiology (STROBE) [25]. The participation of the fighters was voluntary and the responses to the questionnaire were anonymous; all signed the Informed Consent Form (ICF) agreeing to participate in the research, following the ethical principles of Resolution No. 466 of 2012 of the National Health Council (CNS nº466/2012) [26] and the Declaration of Helsinki [27], which stipulate that the individual needs to know exactly what they are getting involved in and freely agree.

An observational, cross-sectional study was conducted, and data collection took place in October and November 2024 in the municipality of Vitória, capital of Espírito Santo. The city has 322,869 inhabitants, a population density of 3324.33 inhabitants/km^2^, and is among the municipalities with the highest population growth in the country [28]. Furthermore, it has already been nationally recognized as one of the places with high levels of physical activity due to its natural characteristics, such as beaches, mountains, and green areas, which stimulate the population [29].

For the sample size calculation, a finite universe of 1000 practitioners was considered. Therefore, a 95% confidence interval, a 5% margin of error, and a 50% prevalence were adopted to maximize the sample size, as this represents the greatest possible variability for any prevalence found [30]. The calculated sample size was 278 individuals, plus 10% to account for possible losses.

Only individuals over the age of 18 who practiced any form of combat sports and were present at the gyms at the time of data collection were voluntarily included in the study. Individuals who were absent from sports practice due to illness, injury, or vacation were excluded. The interviews were face-to-face and individual, in Portuguese, conducted by a single interviewer, using a questionnaire developed for this study and based on two others previously published in the scientific literature [9,11]. The selection and adaptation of the items for the questionnaire followed the methodological recommendations for observational research, preserving the conceptual coherence of the original instruments [31]. The questionnaire contained 25 questions, with an average response time of 15 min, addressing sociodemographic data, access to oral health, sport modality, experiences with dental trauma, use of mouthguards, and guidance on the subject.

Statistical analyses were performed using IBM SPSS, version 20 (IBM Corp., Statistical Package for the Social Sciences, Armonk, NY, EUA). Descriptive analysis was conducted using frequency tables, with absolute and relative values, encompassing sociodemographic variables, dental trauma, and mouthguard use. The association between variables was assessed using the non-parametric Chi-square test, and the Odds Ratio (OR) was calculated to compare individuals exposed and not exposed to dental trauma within the same time frame. The significance level adopted was *p* < 0.05.

## 3. Results

In total, 300 individuals were approached. However, 284 martial arts practitioners (trainers, professionals, semi-professionals, and amateurs) from 17 academies agreed to participate in the research, representing 16 losses; however, this did not compromise the calculated sample, since the final number of participants exceeded the initially estimated quantity.

The sociodemographic profile of the sample obtained was, above all, predominantly male (71.48%), aged up to 25 years (26.41%), single (50%), self-declared white (52.46%), with higher education (39.44%), residents of Vitória (81.35%), and access to oral health services in the private sector (53.52%) (Table 1).

Regarding the type of fighting they practiced, the majority responded Jiu-Jitsu (27.82%), followed by Kickboxing (27.11%). The modalities in which individuals suffered the most LDTs were Capoeira (15.79%), Taekwondo (15.38%), and Kung Fu (12.29%) (Table 2).

Of the participants who reported LDTs (6.69%), 47.37% suffered only one episode, with enamel fracture being the most frequent type (36.84%). Regarding the number of teeth affected (52.64%), only one tooth was affected, most frequently the anterior teeth (47.36%). Regarding treatment, 47.37% did not receive emergency intervention, and among those who did, 31.58% were treated by coaches. The majority (36.84%) were not advised to seek oral health services immediately after the trauma, and 42.11% did not seek treatment subsequently. Among those who sought assistance (63.64%), they did so more than three hours after the event. Regarding the immediate course of action in response to the injury (94.74%), no action was taken (Table 3).

Regarding mouthguards, most participants were not using them at the time they suffered dental injury (78.95%) nor during regular wrestling practice (58.45%). Among those who did use the device, half (50%) used it both in training and in competitions. Most recognized the importance of the device (80.63%) and had already received some guidance on its use (65.14%). However, on the topic of “dental trauma,” most (83.45%) had not received any guidance (Table 4).

When correlating the occurrence of dental trauma with age group (Table 5), a significant association was observed (*p* = 0.016). Individuals aged 37 years or older were approximately 3.3 times more likely to suffer traumatic injury compared to those up to 36 years old, corresponding to 10.37% of cases. The same table also shows that no statistically significant association was observed between dental trauma and the other sociodemographic variables.

## 4. Discussion

Data related to the prevalence and incidence of dental trauma in the sports context are still insufficient, and negligence in the management of dental trauma results in pain and mobility, which can culminate in the loss of a tooth. In addition, aesthetic, functional, and morphological alterations are experienced by individuals who suffer the trauma [32]. Thus, a 6.69% incidence of dental trauma was observed among the study participants, a result consistent with other studies involving contact sports and combat sports—although the literature shows wide variation in prevalence rates due to differences in methodology, the sports disciplines evaluated, and the population characteristics investigated [10,11,12,14,15,16,17,18,33]. Even though the sample of individuals who sustained traumatic dental injuries (TDIs) was small, meaning the absolute frequency of trauma was low, this does not diminish the clinical relevance of the findings; the impacts associated with these sports can cause various types of injuries, including severe ones, which could complicate the prognosis of the observed cases.

The highest prevalences of dental trauma occurred in Capoeira (15.79%), Taekwondo (15.38%), and Kung Fu (12.29%). In contrast, no injuries were recorded in practitioners of Karate, Boxing, and Aikido. The differences in prevalence between the modalities may be attributed to cultural factors, intrinsic characteristics of each martial art (duration, intensity, rules), adherence to mouthguards, and the level of guidance on dental trauma [34]. In Swiss Judo, the use of mouthguards was only permitted in 2018 [12] and the absence of blows directed at the head consequently results in a lower incidence of dental injuries [13]. In Brazil, the recommendation or mandatory use of mouthguards varies in each modality. In Judo, for example, it is not required in training and competitions [35]. These findings suggest that rules, technique, and precautionary measures directly influence the frequency and severity of injuries, reinforcing the need for specific preventive strategies.

Upon further analysis of the types of dental trauma, it is observed that enamel fracture (36.84%) was the most prevalent, predominantly affecting anterior teeth (47.36%). These findings are consistent with other studies, one conducted in Brazil and another in Switzerland, which also indicated enamel fracture as the most frequent lesion, with rates of 20.2% and 76.6%, respectively [12,33]. It is worth noting that there are small methodological differences in the questionnaires of other studies, which specify the types of teeth affected, generally central incisors, as observed in the research carried out in Bahia, Brazil [6]. Even in less severe cases, prevention and proper guidance remain essential.

The diversity of trauma types that can occur in combat situations is evident from the research: in addition to enamel fractures, there were instances of concussion (15.79%), subluxation (15.79%), luxation (10.53%), dentin fractures (15.79%), fractures with pulp exposure (15.79%), complex fractures (10.53%), and dental avulsion. The first three types occurred in association with other injuries. These injuries can compromise the periodontal ligament and pulp vitality even in the absence of coronal fractures; dentin fractures and fractures with pulp exposure showed the same incidence rate and a higher risk of bacterial contamination, potentially requiring endodontic treatment. Complex fractures (despite having one of the lowest incidence rates) and, especially, avulsion are considered the most severe injuries [1,2,21,36].

Dental avulsion is considered a serious type of dental trauma and requires proper management. This injury was identified in 15.79% of the fighters, and it is alarming that 94.74% did not take any immediate action. Among those who sought treatment (63.64%), the search occurred more than three hours after the trauma. These findings corroborate studies conducted in Saudi Arabia and in different regions of Brazil, such as Campinas (São Paulo) and Maceió (Alagoas) [11,14,37]. In this case, immediate reimplantation or maintenance in an appropriate moist environment, such as milk or saline solution, is recommended to preserve the periodontal ligament cells. Neglecting these procedures significantly increases the risk of tooth loss [36,38].

This failure in immediate management is directly reflected in the post-trauma care observed in the sample, showing that the majority (47.37%) of athletes did not receive first aid after dental trauma. This is critical, since the IADT emphasizes immediate management as essential for a favorable prognosis of the dental element [1,21,36,37,39]. The individual who most frequently assisted athletes after trauma was the coach (31.58%). Research shows that coaches play an important role in raising awareness among athletes and are frequently present at the time of accidents [15,16]. In this context, a lack of knowledge about personal protective equipment (PPE), such as gloves and masks, and in the face of traumatic injury, can compromise biosecurity, as well as the affected tooth.

In Brazil, it is observed that most gyms and sports centers lack medical support, which hinders the immediate response to traumatic emergencies. Although health teams are present in high-profile competitions and in some specific sports, this reality does not extend to everyday sports activities. This scenario reaffirms the hypothesis that there is a structural deficiency in educational programs aimed at coaches and teachers, professionals who work on the front lines and who should be properly trained to recognize and manage such situations effectively.

In the context of combat sports, the preventive role of mouthguards is crucial, as they absorb and distribute impact forces, thereby preventing and reducing injuries. There are three types: ready-to-use, moldable, and custom-made (Types I, II, and III, respectively). Type I is sold over-the-counter, lacks retention features, and is less effective; Type II requires softening with heat and molding to the dental arch, which can result in fit issues and poor stability; finally, Type III properly covers the dentition and soft tissues, has adequate thickness, and thus offers superior protection [16,17,40,41,42].

This study reveals a paradox: although the majority (80.63%) of athletes understand their importance, 58.45% do not routinely use them in their sporting practice and 78.95% were not using the device when they suffered the traumatic injury. Furthermore, half of the sample (50%) uses them in training and competitions. The critical point seems to lie in adherence to the device and not in misinformation, since 65.14% of the participants had already received guidance about it. The inconsistent use corroborates the scientific literature, which reports adherence between 41% and 60% [15,17]. Therefore, the preventive effectiveness of mouthguards is conditioned not only by knowledge, but also by their consistent application, a factor directly linked to the reduction in the frequency of dental trauma [42,43].

Furthermore, this study identified a potential educational gap, as 83.45% of the athletes had never received guidance on dental trauma, corroborating another study in which 72.6% had also not been given such guidance [11]. This lack of knowledge reverberates in inadequate post-trauma conduct, and overcoming this challenge requires a multifaceted approach, combining public policies with diverse educational strategies. Such strategies should range from health literacy programs to ongoing training aimed at the general public and healthcare professionals, supported by accessible informational materials [44,45,46,47,48]. In this sense, education emerges as a central pillar for integrating theoretical knowledge with preventive practice, positively impacting the reduction in dental trauma.

When correlated, the age group most affected by dental trauma in this study was adults aged 37 years or older (10.37%; *p* = 0.016). This data corroborates another study conducted with Taekwondo athletes in Croatia, which identified a higher incidence of injuries in the older group, with 42% of senior athletes affected, compared to 13% to 25% in younger groups [18]. However, other studies indicate variation regarding the age of practitioners, suggesting that the risk profile depends not only on age, but also on the sport and its characteristics [11,17,33].

Although the proposed objective was achieved, certain limitations must be considered. As this was a cross-sectional study, data were collected at a single point in time, making it impossible to infer cause-and-effect relationships. Furthermore, the low number of trauma cases limited the analysis of associations such as those between trauma and sociodemographic variables, requiring caution in their interpretation. Additionally, the Regional Council of Physical Education, federations, and local gyms did not provide student numbers or names, citing compliance with the General Data Protection Law [49]. Some websites lacked up-to-date information regarding the addresses, contact details, and locations of gyms offering martial arts classes. It is also worth noting that some practitioners declined to participate in the study. These factors may have introduced selection bias. Consequently, the results reflect only a limited population located in the capital of a single state. Future research should evaluate and compare coaches’ knowledge and the frequency of dental trauma across different disciplines and population contexts to allow for the generalization of findings.

## 5. Conclusions

The prevalence of dental trauma among martial arts practitioners was low. Although the associations observed provide relevant information, they should be interpreted with caution due to the small number of cases. The disciplines with the highest incidence of injuries were capoeira and taekwondo, associated with practitioners over the age of 37. Furthermore, adherence to the use of mouthguards proved insufficient, despite awareness of their importance. Guidance regarding dental trauma was found to be inadequate. Thus, the need to maintain and expand investments in public health policies focused on sports is evident.

## Figures and Tables

**Table 1:** Sociodemographic data of practioners.

Characteristic	Number (*n*)	Percentage (%)
**Sex**		
Male	203	71.48
Female	81	28.52
**Age group**		
Up to 25 years	75	26.41
26–36 years	74	26.06
37–44 years	67	23.59
45 years or older	68	23.94
**Marital status**		
Single	142	50.00
Married	108	38.02
Stable union	15	5.28
Separated	3	1.06
Divorced	16	5.64
**Ethnicity**		
White	149	52.46
Black	23	8.10
Brown (mixed race)	101	35.56
Asian	8	2.82
Indigenous	3	1.06
**Education level**		
High school	85	29.93
Higher education	112	39.44
Postgraduate	87	30.63
**City of residence**		
Serra	20	7.04
Vitória	231	81.35
Vila Velha	28	9.86
Cariacica	2	0.70
Viana	1	0.35
Guarapari	1	0.35
Domingos Martins	1	0.35
**Type of access to oral health services**		
No access	5	1.76
Public health system (SUS)	33	11.62
Private	152	53.52
Health insurance	94	33.10
Total	284	100.00

**Table 2:** Contact sports practiced and their relationship with traumatic dental injuries.

Sport	Number (%)	Number With Trauma (%)	Number Without Trauma (%)
Jiu-Jitsu	79 (27.82)	8 (10.13)	71 (88.97)
Judo	33 (11.62)	2 (6.06)	31 (93.94)
Karate	19 (6.69)	0 (0.00)	19 (100.00)
Taekwondo	13 (4.58)	2 (15.38)	11 (84.62)
Boxing	11(3.87)	0 (0.00)	11 (100.00)
Kickboxing	77 (27.11)	3 (3.90)	74 (96.10)
Capoeira	19 (6.69)	3 (15.79)	16 (84.21)
Krav Magá	26 (9.15)	1 (3.85)	25 (96.15)
Muay Thai	25 (8.80)	3 (12.00)	22 (88.00)
Aikido	8 (2.82)	0 (0.00)	8 (100.00)
Kung Fu	7 (2.46)	1 (12.29)	6 (85.71)

Percentages exceed 100% because participants could select more than one option.

**Table 3:** Dental trauma data among contact sports practitioners and attitudes taken after traumatic dental injury.

Characteristic	Number (%)	Characteristic	Number (%)
**Experienced dental trauma during practice**		**Received assistance**	
No	265 (93.31)	Coach	6 (31.58)
Yes	19 (6.69)	Teammate	2 (10.53)
**Number of trauma episodes**		Physician	1 (5.26)
One	9 (47.37)	Dentist	1 (5.26)
Twice	6 (31.58)	No one	9 (47.37)
Three or more times	4 (21.05)		
**Type of dental trauma (multiple answers allowed)**		**Advised to seek dental care**	
Concussion	3 (15.79)	Yes	7 (36.84)
Subluxation	3 (15.79)	No	12 (63.16)
Luxation	2 (10.53)	**Service sought after trauma**	
Enamel fracture	7 (36.84)	Public health unit	1 (5.26)
Dentin fracture	3 (15.79)	Private dentist	7 (36.84)
Fracture with pulp exposure	3 (15.79)	Dentist through insurance	3 (63.16)
Dental avulsion	3 (15.79)	Did not seek care	8 (42.11)
Complex fractures	2 (10.53)	**Time to seek care after trauma**	
**Number of affected teeth**		Immediately	1 (9.09)
One	10 (52.64)	30 min	1 (9.09)
Two	3 (15.79)	1 h	2 (18.18)
Three	4 (21.05)	More than 3 h	7 (63.64)
More than three	2 (10.52)		
**Type of affected tooth**		**Immediate action after trauma**	
Anterior	9 (47.36)	Stored in water	1 (5.26)
Posterior	5 (26.32)	None	18 (94.74)
Anterior and posterior	5 (26.32)		

Percentages for trauma type exceed 100% because multiple answers were allowed.

**Table 4:** Mouthguard use and guidance regarding mouthguards and traumatic dental injuries.

Characteristic	Number (%)
**Use of mouthguard**	
No	166 (58.45)
Yes	118 (41.55)
**Situation in which mouthguard is used**	
Training and competitions	59 (50.0)
Competitions only	12 (10.17)
Training only	47 (39.83)
**Believes mouthguard use is important**	
Yes	229 (80.63)
No	55 (19.37)
**Received guidance about mouthguard use**	
Yes	185 (65.14)
No	99 (34.86)
**Received guidance about dental trauma**	
Yes	47 (16.55)
No	237 (83.45)
**Used a mouthguard when the trauma occurred**	
Yes	4 (21.05)
No	15 (78.95)

**Table 5:** Association between traumatic dental injuries and sociodemographic variables.

Characteristic	With Trauma (number)	With Trauma %	Without Trauma (number)	Without Trauma %	*p*-Value	OR 95% CI
**Sex**						
Male	17	8.37	186	91.63	0.055	3.610
Female	2	2.47	79	97.53		0.815–15.997
**Age group**						
Up to 36 years	5	3.36	144	96.64	0.016 *	3.333
37 years or older	14	10.37	121	89.63		1.167–9.524
**Marital status**						
Without spouse	10	6.21	151	93.79	0.445	1.192
With spouse	9	7.32	114	92.68		0.469–3.030
**Education level**						
High school	6	7.06	79	92.94	0.526	1.087
Higher education or postgraduate	13	6.53	186	93.47		0.399–2.961
**Access to oral health care**						
No access	0	0.00	5	100.00	0.706	-
SUS/Insurance/Private	19	6.81	260	93.19		-

OR: Odds Ratio; CI: Confidence Interval; * *p*-value < 0.05 indicates statistical significance.

## Data Availability

The original contributions presented in this study are included in the article. Further inquiries can be directed to the corresponding authors.
